# Using REGARDS to Study Family Caregiver Well-Being, Health, and Mortality

**DOI:** 10.1093/geroni/igaa057.3153

**Published:** 2020-12-16

**Authors:** David Roth, William Haley

**Affiliations:** 1 Johns Hopkins University, Baltimore, Maryland, United States; 2 University of South Florida, Tampa, Florida, United States

## Abstract

The REGARDS study has provided a unique opportunity to study both disease-specific (stroke) and broader samples of family caregivers, and to examine the effect of transitions to caregiving over time. Using REGARDS has afforded many advantages over conventional caregiving research, including the availability of biomarker and mortality data, a large sample of non-caregiving controls who can be carefully matched to caregivers, and ability to track onset of caregiving over time. Our findings illustrate the complex nature of caregiving-related effects. While caregiving leads to worse psychological well-being, we have found minimal physical health decreases and reduced mortality rates compared to matched non-caregiving samples. These findings have policy implications and have challenged the conventional beliefs about caregiving based on previous studies of convenience samples. Diverse students and junior faculty members from multiple universities have also gained experience and contributed to high impact papers from this work.

